# Assessing the Effect of Flour (White or Whole-Grain) and Process (Direct or Par-Baked) on the Mycotoxin Content of Bread in Spain

**DOI:** 10.3390/foods12234240

**Published:** 2023-11-24

**Authors:** Manuel Gómez, Andrea Casado, Irma Caro

**Affiliations:** 1 Food Technology Area, College of Agricultural Engineering, University of Valladolid, 34071 Palencia, Spain; andrea.c.f@hotmail.com; 2 Food Science and Nutrition, Faculty of Medicine, University of Valladolid, 47005 Valladolid, Spain; irma.caro@uva.es

**Keywords:** bread, whole grain, artisan, par-baked, deoxynivalenol, ochratoxin, aflatoxin

## Abstract

Bread is the staple food in many parts of the world. Like other foods, bread can contain mycotoxins resulting from microbial development throughout the supply chain (from field to table). In this study, baguette-style bread from small artisanal bakeries (direct) and supermarkets (par-baked loaves made by large companies) in Castile and Leon (Spain) was analyzed. Both white and whole-grain breads were collected from all retail outlets. The mycotoxins analyzed included deoxynivalenol (DON), ochratoxin (OTA), and aflatoxin B1 and B2 (AFB1, AFB2). All of the bread samples studied had mycotoxin levels below the maximum limits established by legislation. The presence of DON was higher than that of OTA, and AFB1 and AFB2 could not be quantified. Industrial breads had higher levels of DON and OTA (only in the whole-grain breads) compared to artisanal breads. However, no significant differences were found between white and industrial breads beyond those mentioned above. These results demonstrate that the established control chains ensure low mycotoxin content in bread of this type.

## 1. Introduction

Mycotoxins are naturally occurring secondary metabolites produced by various genera of molds, such as Aspergillus, Fusarium, Penicillium, and Alternaria. About 400 mycotoxins have been identified, with some of the most important ones being aflatoxins B1 (AFB1), aflatoxin B2 (AFB2), Ochratoxin (OTA), and deoxynivalenol (DON) [1]. The consumption of mycotoxin-contaminated foods can have serious health consequences, being potentially fatal, depending on the specific mycotoxin and the amount consumed. Harmful effects include hepatotoxicity, nephrotoxicity, carcinogenesis, immunosuppression, and mutagenicity [2]. Although it is possible to develop acute mycotoxicosis after exposure to large amounts of mycotoxins, which manifests itself rapidly, one of the main problems of mycotoxin exposure is related to its chronic toxicity. Thus, people may suffer adverse health effects after long-lasting exposure to a low dose of mycotoxins. The symptoms associated with this form of mycotoxicosis will depend on the amount and duration of exposure, and other factors such as the age, sex, health, diet, or genetics of the individual [3].

Depending on the cultivation and storage conditions, cereals may suffer from microbial contamination, resulting in mycotoxin contamination. In this way, the intake of cereals and cereal products can be one of the major sources of mycotoxins in the diet. Thus, studies on the mycotoxin content of cereals, and measures to reduce it, are very numerous and have given rise to extensive reviews [4,5]. Approximately 25% of cereals globally are contaminated with mycotoxins [6], depending on the cultivation and storage conditions. According to Yu and Pedroso [5], in developing countries, mycotoxin contamination of cereals poses a serious risk to human health due to insufficient enforcement of food safety regulations and food shortages. While in developed countries the risk is lower for adults, it is still an appreciable risk factor for infants and young children.

The problems associated with climate change should also be highlighted. The progressive warming of the planet has led to greater microbial contamination of crops in certain parts of the world in recent years, associated with an increased presence of mycotoxins. Forecasts suggest that this problem may increase in the coming decades [7].

Bread is a dietary staple food in many regions of the world, and therefore, its mycotoxin content can have a significant impact on the population. Thus, studies on the mycotoxin content of bread are most numerous among cereal derivatives, after those focused on cornflakes. Among the countries where most studies have been carried out, some of the most developed countries stand out, such as Germany [4]. In fact, studies carried out in Portugal [8] and Morocco [9] highlight the problem of mycotoxin contamination in bread as one of the main sources of these contaminants in the diet of these countries.

A mixture of flour, water, salt, and yeast, occasionally accompanied by other ingredients, is kneaded, fermented, and baked to make bread. The mycotoxin content of flour will depend mainly on the mycotoxin content of the grains used in the milling process. It is true that practices to reduce the mycotoxin content are possible in the milling process, especially in whole-meal flours, but they do not eliminate mycotoxins completely. In this way, mycotoxins present in the flour will find their way into the dough. It should also be noted that the mycotoxin content is typically higher in whole-grain flours compared to white flours [10], which could influence the mycotoxin content of bread made from these flours. At this point, it is possible to achieve a reduction in the mycotoxin content through the action of certain microorganisms (fermentation) and the application of high temperatures (baking). However, this will depend on the conditions of these processing steps, and in no case can they achieve the complete elimination of mycotoxins, but only a partial reduction [11,12]. Indeed, mycotoxins are one of the major contaminants in bread, and pose a potential risk to human health [13].

Nowadays, it is common to find baked bread sold in supermarkets from industrial par-baked bread. In this process, par-baked breads are partially baked and frozen for distribution to retail outlets. In this case, time–temperature combinations are not as high as in traditional baking, which may reduce the mycotoxin-reducing effect of this processing step. In fact, Vidal et al. [14] found the baking time to be more important than the baking temperature for the reduction in DON content. Par-baked doughs also have high water activity, a higher risk of microbial growth [15] and therefore a higher risk of mycotoxin contamination if not handled properly.

Additionally, molds can grow and produce mycotoxins in bread if the storage conditions, like the water activity and temperature, are appropriate [16]. However, there are also breads with lean formulas and very crispy crusts, which are discarded 24 h after baking due to the loss of these qualities. In these cases, microbial growth during the shelf life is minimal, and so is the associated mycotoxin content. However, this short shelf life generates a large amount of food waste that needs to be minimized, which requires that there are no food safety problems [17].

Previous studies have investigated the presence of certain mycotoxins in commercial bread from various regions around the world, including Spain [18,19,20], Germany [21], France [22], China [23], and Portugal [24]. However, none of these studies have assessed the influence of the type of wheat flour used (white or whole-grain) or the bread-making process (industrial par-baked or artisanal) on these mycotoxin levels.

In this study, bread samples of the most consumed type in Spain (baguette) were collected from 10 artisanal bakeries and 10 supermarkets, where bread is baked on-site using par-baked bread. In both cases, bread made from white flour and wholegrain flour was sampled. The collected bread samples were analyzed for their DON, OTA, and Aflatoxin B1, and B2 contents.

## 2. Materials and Methods

### 2.1. Materials

#### 2.1.1. Breads

**Figure 1:**
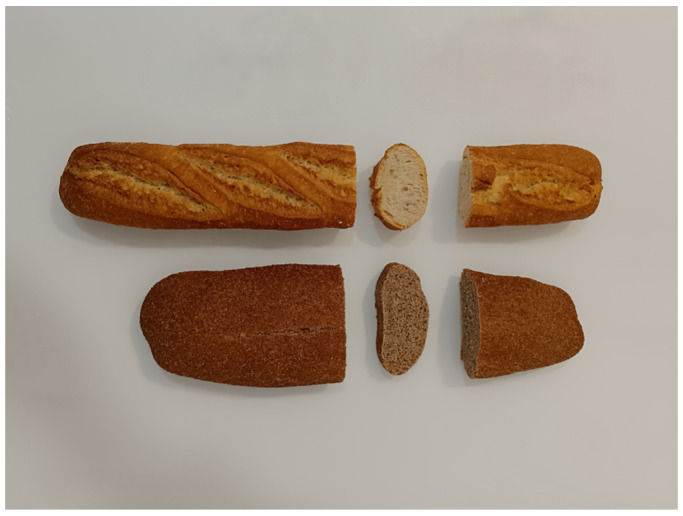
Picture of white (**above**) and whole-grain (**below**) breads.

Breads were purchased from 10 artisanal establishments, which produced their bread from flour on the same day of sale. Additionally, bread was purchased from 10 supermarkets or hypermarkets that baked par-baked bread, which was manufactured by large industries for retail purposes. All the breads were of the type most consumed in Spain, ‘barra’, similar to the French baguette, weighing approximately 250 g. These breads are made with lean formulas (flour, water, salt, yeast, and bread improvers) and have a crispy crust and a soft crumb. In no case were the establishments informed in advance or provided with information regarding the ongoing study. In all of the establishments, samples of both white bread and whole-grain bread (made entirely from whole wheat flour) were purchased (Figure 1). In all establishments, all types of bread were purchased on two separate days (one replicate), with a 4-week interval between purchases. All samples were acquired between April and July 2023 in Castile and Leon, Spain (León, Palencia, or Valladolid provinces).

#### 2.1.2. Chemicals Reagent

The OTA, DON and aflatoxin B1 and B2 standards (purity > 99%) were supplied by Romer Labs (Getzersdorf, Austria). Acetonitrile and methanol were of HPLC-grade, obtained from VWR BDH Chemicals (West Chester, PA, USA), and purified water (<18 MΩ·cm of resistivity) was delivered using a Millipore-Q water purification device (Bedford, MA, USA).

### 2.2. Methods

#### 2.2.1. Preparation of Sample

Bread samples were transported to the laboratory in the container provided by the processor and processed within 24 h. Half of each loaf of bread, including the crumb and crust, was milled using a kitchen processor (Cecotec 1000; Valencia, Spain), transferred to a plastic bag, and stored at −20 °C until the extraction procedure within 24 h.

#### 2.2.2. Extraction Procedure

The extraction of mycotoxins was performed according to Vaclavikova et al. [25] with some modifications, as briefly described below. Two grams of the ground bread was added to 7.5 mL of Milli-Q H_2_O and 10 mL of acetonitrile. The mixture was shaken vigorously by hand for 3 min. After that, 1 g of NaCl and 4 g of MgSO_4_·1H_2_O were added to the reaction and shaken once again under the same conditions. After that, the samples were centrifuged for 5 min at 5000 rpm and 5 °C. The organic phase was obtained with a Pasteur pipette, added into a Falcon tube and vortexed for 1 min. The organic phase was stored at −18 °C until analysis. Then, 1 mL of organic phase was introduced into the centrifuge tube (Eppendorf, Hamburg, Germany) and was dried totally with nitrogen gas at 35 °C using a dry-block heater (Reacti-ThermI ™ #TS-18822) supplied by Thermo Scientific™ (Waltham, MA, USA). After this, 1 mL of MeOH-H_2_O (1:1) was used to suspend the dry sample. Finally, the mixture was shaken and filtrated through membrane nylon with a pore of 0.22 µm and 13 mm of diameter (SFNY-122-100, BRANCHIA) before the analysis.

#### 2.2.3. LC-MS/MS Parameters

Detection and quantification of mycotoxins were performed using an ultra-high-performance liquid chromatography (UHPLC) Sciex Exion system connected to a Sciex 6500 + triple quadrupole mass spectrometer from Sciex (Washington, DC, USA) equipped with an electrospray ionization (ESI) source operated in a positive mode. Chromatographic separation was carried out using a Phenomenex (Washington, DC, USA) polar column Luna Omega Polar C18 (2.1 mm × 100 mm, particle size 1.6 µm), which was temperature-controlled at 40 °C during the separation process. The gradient was run at 0.4 mL min^−1^ using water containing 0.1% of formic acid (*v*/*v*) and acetonitrile containing 0.1% of formic acid (*v*/*v*) as mobile phases. The gradient program started with 20% of the organic phase for 1 min and then we increased the organic phase to 90% in 5 min, held it at 90% for 2 min, and returned it to the initial conditions (20%) for 0.1 min, holding it at 20% for 5.9 min. The total chromatography time was 12 min. The column was kept at 40 °C, and samples were preserved in the autosampler at 15 °C. The injection volume was 10 µL. 

Mass spectrometry acquisition was performed using the triple quadrupole Sciex 6500+QqQ in selected reaction monitoring (SRM) mode, recording the transitions between the precursor ion and the two most abundant product ions for each target mycotoxin. 

The ESI was used to identify and quantify mycotoxins, and the operation parameters were as follows: source temperature, 300 °C; capillary voltage, 4.5 kV (positive); N_2_ was used at 35 psi for curtain gas, both ion source gas 1 and 2 were used at 45 psi, and the collision gas was used at 9.0 psi. To achieve the optimum mass sensitivity/selectivity ratio, the unit resolution was set at both the first (Q1) and third (Q3) quadrupole. Each of the daughter ion and mass spectrometry instrument conditions for the target mycotoxins are presented in Table 1. Instrument control and data acquisition were performed using Analyst^®^ software (Sciex, version 1.7.3). Data processing was carried out using SciexOS software (Sciex, version 1.4.1). 

External standard calibration curves were performed for each mycotoxin by plotting the signal intensity versus the mycotoxin concentration. The coefficients of determination (R^2^) of the linear regression models obtained were higher than 0.9990. The detection limits (LOD) and quantification limits (LOQ), calculated by analyzing blank samples spiked with standard mycotoxins in quintuplicate as the lowest concentration of mycotoxin that produces a chromatographic peak at a signal-to-noise ratio (S/N) of 3 and 10, respectively, were as follows for LOD (μg/kg): 0.038, AFB1; 0.025, AFB2; 0.5, DON, and 0.018, OTA; and for LOQ (μg/kg), they were as follows: 0.115, AFB1; 0.075, AFB2; 5.0, DON; and 0.055, OTA. 

The detection and quantification of mycotoxins were carried out at the Laboratory of Instrumental Techniques (LTI) of the University of Valladolid.

#### 2.2.4. Statistical Analysis

Analysis of variance (ANOVA) was used to study the differences between types of bread and the amount of mycotoxin. Tukey’s HSD was used to describe means with 95% confidence. The analysis was performed using Statgraphics Plus V5.1 software (Statpoint Technologies, Warrenton, VA, USA).

## 3. Results and Discussion

Table 2 displays the mycotoxin content values for different types of bread. Table 3 shows the mycotoxin content of bread collected at the different points of sale. Notably, AFB2 was not detected, and the AFB1 content in all samples was below the detection limit. This may explain why most studies on the mycotoxin content of wheat and bread have focused on analysing DON and OTA [12]. As for DON and OTA values in all cases, they were significantly lower than the legal limits set by the European Union (500 μg/kg for DON and 3 μg/kg for OTA) [26]. Therefore, these values are not a cause for concern. This confirms the food safety of these bread types and their potential reuse within the food chain, at least in terms of mycotoxin content.

Our findings are also consistent with previous results, such as those of Sirot et al. [22], who did not observe the presence of aflatoxins but did find DON and OTA. In general, the values observed in our study and the percentage of positive samples are slightly lower than those found in the study by Sirot et al. [22] and by Jiang et al. [23] for steamed bread, and Schollenberger et al. [21] for German bread. These differences may be attributed to the type of bread, as steamed bread lacks a protective crust, and German bread, with a high presence of rye flour, has a longer shelf life than Spanish baguettes, which may lead to a slightly higher risk of microbial growth. On the other hand, the results obtained by González-Osnaya et al. [19] for bread purchased in Valencia, Spain, were similar to ours but with greater variability. They found a lower number of positive samples but, in some cases, higher values than those observed in our study. This may be due to a wider variety of bread types studied, ranging from breadcrumbs or toasted bread to sandwich loaves, with an extended shelf life.

In our study, DON and OTA values are higher in industrial bread compared to artisanal bread, with the difference being more pronounced in the case of DON, where industrial bread triples the content of artisanal bread. Additionally, a higher percentage of samples have been identified with this mycotoxin, especially in whole-grain bread, where it is detected in 100% of industrial bread and only 50% of artisanal bread. Differences between bread made from white or whole-grain flour are smaller, and significant differences are observed only for OTA, with higher values in whole-grain bread, specifically in industrial bread. A similar pattern emerges in the percentage of samples with positive results, slightly higher in whole-grain bread compared to white bread, especially for industrial bread. 

The evaluated bread types (baguettes) have a very short shelf life, as the crust, valued for its crispness, quickly becomes chewy. To better preserve this crunchy characteristic, they are not stored or sold in sealed containers, which leads to faster drying and hardening of the crumb. For this reason, they are typically removed from sale within 24 h of being made. Therefore, it is not common to encounter significant microbial growth, and consequently the potential for mycotoxin development, between their production and sale. In fact, a previous study confirmed the low microbial content of these bread types at the time of their removal from sale [27]. Hence, the source of mycotoxin content in these bread types should be sought in the raw materials or the production processes prior to baking.

It should be noted that bread made with whole wheat flour shows minimal differences with respect to bread made with white flour in terms of the average mycotoxin content. Various studies have demonstrated higher DON content in the outer parts of the grain (bran) compared to white flour or in whole-grain flours compared to white flours [28,29,30]. A similar trend is observed for OTA content [31]. It is true that during the bread-making process, especially during baking, there can be some reduction in mycotoxin content, particularly for DON [32] and to a lesser extent for OTA [33], as it is more heat-stable. However, this alone cannot explain the minimal differences found in our study between white and whole-grain bread. It is necessary to consider that studies on differences between white and whole-grain or bran flours were conducted in regions or periods with microbial contamination issues in wheat, or the samples were contaminated after harvesting, unlike the situation in Spain or in countries from which Spain imports wheat, such as France, the USA, or Canada, where there is a lower risk of microbial development. Additionally, practices in modern milling industries, which avoid introducing contaminated wheat through stringent quality controls, should be considered. Some practices such as thorough grain cleaning can also reduce mycotoxin content [34]. A gentle pearling process can also help to reduce mycotoxin levels in the outer layers or whole-grain flour [35].

In fact, our results align with those of Saladino et al. [20], who found minimal differences between white and whole-grain bread in a study on aflatoxin in packaged commercial bread.

The higher mycotoxin content in bread made from industrial par-baked products compared to artisanal ones may also catch one’s attention, as many consumers believe that the industry maintains a higher level of hygiene and controls than smaller artisanal businesses. Several possible explanations exist for these differences. On the one hand, we can consider that manufacturers of precooked bread require stronger flours and, therefore, a higher percentage of imported wheat for flour production. Since Spanish wheat is not prone to microbiological contamination due to climate conditions, mycotoxin content might originate from these imported wheat sources. In fact, in the study on flour type, the only differences between whole grain and white bread were detected in the industrial bread samples. However, the disparities between artisanal and industrial bread are more pronounced, and the controls conducted in countries like Spain and the international trade of wheat for human consumption should reduce these risks. Therefore, we must consider alternative explanations.

A second explanation revolves around the fact that after the initial baking, par-baked bread retains high moisture levels, and therefore it is a product with high water activity. In addition, the heat treatment received in the initial baking is not sufficient to “sterilize” the doughs, and if not cooled rapidly, accelerated microbial development can occur to a much greater extent than in finished bread [36]. The same can happen once they are thawed before the second baking or during storage or transportation if there is a breakdown in the cold chain. Thus, there are several points in the process with a high risk of microbial growth that do not exist in artisanal bread production, where the process is continuous and, after fermentation, the bread undergoes baking and “sterilization”. It is assumed that companies are aware of this issue and have measures in place to prevent it, but the risk still exists.

A third possible explanation is the practice of reusing defective products. There is a trend to reduce food waste, and the practice of incorporating remnants of defective bread into new batches has been proposed [17]. In the production plants of par-baked bread, reintroducing defective loaves—for example, those with a low volume or with small proportions—into the production of new doughs is common. This is simpler than in other types of bread production, because the heat treatment is shorter, there are fewer changes in the dough, especially on the outer surface, where Maillard and caramelization reactions do not fully develop, and the colour remains unchanged. If these defective loaves are not stored properly until reintroduction, the risk of microbial development is very high, and therefore the risk of generating mycotoxins is also high [17]. Hence, while proper practices can mitigate these dangers, the production of par-baked bread carries more risks for microbial development and mycotoxins than the production of artisanal bread.

## 4. Conclusions

The mycotoxin content in the most consumed bread type in Spain (baguettes) is significantly below the limits established by legislation in all analyzed cases. It is noteworthy that there are minimal differences between breads made from white flour and whole grain flour, and that bread produced in supermarkets or hypermarkets, using par-baked bread as a starting material, exhibits higher mycotoxin levels compared to artisanal bread directly prepared in small bakeries. These findings are reassuring for consumers who can safely consume this type of bread and for the potential reuse of these bread products in the human food chain. In fact, artisanal bread is often more susceptible to waste, as it is typically produced at the beginning of the day and may be prepared in excess to meet possible demand peaks. Furthermore, this serves as a signal to exercise increased caution during the production of par-baked bread and its final processing at retail points, given the observed higher risk. However, the limited number of bakeries (10) and hypermarkets (10) visited has to be taken into account, so it is necessary to increase the number of establishments studied in future studies in order to obtain more general conclusions.

## Figures and Tables

**Table 1:** List of SRMs and mass spectrometry instrument conditions for the target mycotoxins.

RT (min)	Mycotoxin Ions	Q1 (*m*/*z*)	Q3 (*m*/*z*)	Transition Ion	DP (V)	CE (V)	CXP (V)	Dwell (ms)
8.55	OTA pos 1	404.10	239.00	Q_t_	45.00	28.00	19.00	50.00
	OTA pos 4		221.00	Q_f_	45.00	60.00	19.00	50.00
1.08	DON pos 1	297.11	249.20	Q_t_	46.00	15.00	10.00	50.00
	DON pos 3		203.20	Q_f_	46.00	25.00	22.00	50.00
7.18	AFB1 pos 1	312.97	285.20	Q_t_	106.00	35.00	10.00	50.00
	AFB1 pos 2		241.00	Q_f_	106.00	53.00	18.00	50.00
6.81	AFB2 pos 1	315.02	287.10	Q_t_	116.00	37.00	10.00	50.00
	AFB2 pos 2		259.10	Q_f_	116.00	43.00	8.00	50.00

Q: mass of pseudion; Qt: quantifier ion; Qf: qualifier ion; DP: entry orifice potential; CE: collision energy; CXP: collision cell exit potential.

**Table 2:** DON and OTA values as a function of the flour and the process in artisanal and industrial bread.

Samples	n	DON (μg/kg)				OTA (μg/kg)			
		Positive Samples and Frequency (%)	DON			Positive Samples and Frequency (%)	OTA		
			Media	Max.	Min.		Media	Max.	Min.
White Artisanal	20	8 (40%)	12.02 ± 19.58 ^b^	73.84	<LOD	11 (55%)	0.09 ± 0.10 ^b^	0.37	<LOD
White Industrial	20	14 (70%)	31.05 ± 33.96 ^a^	99.52	<LOD	11 (55%)	0.07 ± 0.07 ^b^	0.20	<LOD
Whole-Grain Artisanal	20	10 (50%)	13.49 ± 16.45 ^b^	50.29	<LOD	11 (55%)	0.08 ± 0.13 ^b^	0.77	<LOD
Whole-Grain Industrial	20	20 (100%)	42.80 ± 21.32 ^a^	91.19	5.87	16 (80%)	0.19 ± 0.15 ^a^	0.64	<LOD

The values with the same letter in the same column do not present significant differences (*p* < 0.05). <LOD, lower than the detection limit.

**Table 3:** Mean OTA and DON values for the breads purchased at each point of sale.

Point of Sale	OTA		DON	
	White	Whole-Grain	White	Whole-Grain
I1	<LOD	0.25 ± 0.06 ^ABC^	16.55 ± 11.93 ^CD^	88.77 ± 3.08 ^A^
I2	0.09 ± 0.10 ^BC^	0.14 ± 0.01 ^BCD^	8.56 ± 1.33 ^CD^	54.46 ± 1.83 ^BC^
I3	0.11 ± 0.01 ^B^	0.16 ± 0.01 ^BCD^	23.02 ± 8.07 ^C^	33.69 ± 1.50 ^DEF^
I4	0.16 ± 0.04 ^B^	0.25 ± 0.09 ^ABC^	<LOD	56.63 ± 11.42 ^B^
I5	0.12 ± 0.01 ^B^	0.31 ± 0.01 ^AB^	90.14 ± 6.95 ^A^	50.07 ± 3.52 ^BCD^
I6	<LOD	<LOD	<LOD	35.10 ± 6.46 ^CDEF^
I7	0.13 ± 0.04 ^B^	0.47 ± 0.19 ^A^	71.66 ± 15.31 ^AB^	34.70 ± 6.18 ^CDEF^
I8	<LOD	<LOD	<LOD	12.07 ± 8.02 ^GHI^
I9	0.12 ± 0.01 ^B^	0.20 ± 0.11 ^BCD^	25.63 ± 0.98 ^C^	40.85 ± 11.39 ^BCDE^
I10	<LOD	0.11 ± 0.01 ^BCD^	74.93 ± 11.73 ^AB^	21.55 ± 3.21 ^EFGH^
A1	0.33 ± 0.05 ^A^	0.29 ± 0.32 ^ABC^	61.31 ± 10.05 ^B^	6.25 ± 7.23 ^HI^
A2	0.08 ± 0.09 ^BC^	<LOD	13.10 ± 15.18 ^CD^	41.05 ± 1.17 ^BCDE^
A3	<LOD	0.13 ± 0.07 ^BCD^	14.61 ± 5.29 ^CD^	15.54 ± 17.98 ^FGHI^
A4	<LOD	0.13 ± 0.04 ^BCD^	<LOD	26.73 ± 2.74 ^EFG^
A5	0.08 ± 0.03 ^BC^	<LOD	14.63 ± 1.27 ^CD^	34.16 ± 16.42 ^DEF^
A6	<LOD	<LOD	<LOD	<LOD
A7	<LOD	0.06 ± 0.07 ^CD^	<LOD	<LOD
A8	0.16 ± 0.01 ^B^	<LOD	<LOD	5.21 ± 6.01 ^HI^
A9	0.14 ± 0.03 ^B^	0.09 ± 0.03 ^BCD^	<LOD	5.93 ± 6.85 ^HI^
A10	0.14 ± 0.01 ^B^	0.14 ± 0.02 ^BCD^	16.57 ± 19.37 ^CD^	<LOD

I, samples from industrial processing; A, samples from artisanal processing. The values with the same letter in the same column do not present significant differences (*p* < 0.05). <LOD, lower than the detection limit.

## Data Availability

The data used to support the findings of this study can be made available by the corresponding author upon request.
